# Christian Science and Diphtheria; Important Decision

**Published:** 1903-11

**Authors:** 


### Christian Science and Diphtheria; Important Decision.

The New York Court of Appeals (the court of last resort in that State) has just sustained a decision of the lower courts in a matter of vital interest to the medical profession. The verdict from which appeal was made had subjected defendant to a fine of $500, or 500 days in prison for neglecting to call in "competent medical assistance" in the case of an adopted daughter, who recently died of pneumonia. The court held that "it would seem that the legislative intent is reasonably clear, although possibly more concise language could have been employed. The section of the code under which the indictment was found contemplates that there are persons on whom the law casts a duty of caring for minors. Sitting as a court of law for the purpose of construing and determining the meaning of statutes, we have nothing to do with variances in religious belief, and have no power to determine which is correct. We place no limitation on the power of the mind over the body, the power of faith to dispel disease, or the power of the Supreme Being to heal the sick. We merely declare the law as given us by the Legislature. We find no error on the part of the trial court which calls for a reversal."

The lower court had held that in as much as defendant was entrusted with the care and safety of the child, he must in case of illness call in "competent medical assistance," if, in his judgment, the severity of the case demanded it. In defining this term the court held the opinion that only such persons as were licensed by the State—regulars, homeopaths, eclectics, etc.—were capable of rendering such assistance in the meaning of the law. Believing this case to be a typical one, the adherents of "faith cure," Christian Science," etc., from the country at large, subscribed liberally to obtain competent advocates, fight the decision in the upper courts of the State, and secure if possible a favorable decision. The result was as above stated.

Dowie threatens to carry the case to the Supreme Court of the United States. If this is done, the result will be watched with interest. If no such appeal is made, the decision certainly creates a precedent.

